# Targeting Sterylglucosidase A to Treat Aspergillus fumigatus Infections

**DOI:** 10.1128/mbio.00339-23

**Published:** 2023-03-06

**Authors:** Nivea Pereira de Sa, Kalani Jayanetti, Dominick Rendina, Timothy Clement, Veronica Soares Brauer, Caroline Mota Fernandes, Iwao Ojima, Michael V. Airola, Maurizio Del Poeta

**Affiliations:** a Department of Microbiology and Immunology, Stony Brook University, Stony Brook, New York, USA; b Department of Chemistry, Stony Brook University, Stony Brook, New York, USA; c Institute of Chemical Biology and Drug Discovery, Stony Brook, New York, USA; d Department of Biochemistry and Cell Biology, Stony Brook University, Stony Brook, New York, USA; e Division of Infectious Diseases, School of Medicine, Stony Brook University, Stony Brook, New York, USA; f Veterans Administration Medical Center, Northport, New York, USA; University of Florida College of Dentistry

**Keywords:** sterylglucoside, sterylglucosidase, antifungal, *Aspergillus fumigatus*

## Abstract

Invasive fungal infections are a leading cause of death in immunocompromised patients. Current therapies have several limitations, and innovative antifungal agents are critically needed. Previously, we identified the fungus-specific enzyme sterylglucosidase as essential for pathogenesis and virulence of Cryptococcus neoformans and Aspergillus fumigatus (*Af*) in murine models of mycoses. Here, we developed *Af* sterylglucosidase A (SglA) as a therapeutic target. We identified two selective inhibitors of SglA with distinct chemical scaffolds that bind in the active site of SglA. Both inhibitors induce sterylglucoside accumulation and delay filamentation in *Af* and increase survival in a murine model of pulmonary aspergillosis. Structure-activity relationship (SAR) studies identified a more potent derivative that enhances both *in vitro* phenotypes and *in vivo* survival. These findings support sterylglucosidase inhibition as a promising antifungal approach with broad-spectrum potential.

## INTRODUCTION

Aspergillus fumigatus (*Af*) is a saprotrophic fungus ubiquitously found in the environment that, upon inhalation, causes both acute and chronic illnesses in at-risk individuals (1). It is estimated that humans inhale hundreds of *Af* conidia every day that readily reach the alveolar spaces due to their relatively small size (2 to 3 μm), indicating a constant daily battle at the host-pathogen interface in the upper respiratory tract and lower airways (2, 3). Healthy individuals exposed to *Af* mount an appropriate immune response resulting in pulmonary clearance of the fungus (4). However, individuals with compromised immunity fail to control the inhaled conidia, which germinate to hyphae upon entering the lung parenchyma, causing invasive disease. In addition, injury to the respiratory tract stimulates fungal invasion and the emergence of disease—for example, that caused by severe acute respiratory syndrome coronavirus 2 (SARS-CoV-2) (5) can lead to coronavirus disease 2019 (COVID-19)-associated pulmonary aspergillosis (6, 7). The incidence of invasive aspergillosis due to *Af* has increased 3-fold in the last decade (8), and its mortality has risen by over 300% (9). The Infectious Diseases Society of America listed *Af* as one of six pathogens for which a substantive treatment breakthrough is urgently needed (10). Recently, the World Health Organization (WHO) listed the fungal priority pathogens, including *Af*, in the critical priority group, for which research and new treatments are most needed due to its public health burden (11).

The classic polyene antifungal amphotericin B deoxycholate was discovered over 50 years ago, and the triazole antifungals were approved in the early 1990s. The second-generation triazoles, voriconazole and posaconazole, expanded the antifungal spectrum but were only pharmacologic moiety modifications and suffer many of the same drawbacks and resistance pitfalls as their predecessors. The echinocandin antifungals, which act on the fungal cell wall, were developed in the mid-1990s and approved for use in the early 2000s (12). Thus, the growing number of patients with invasive fungal infections has greatly outpaced antifungal development, at least in part due to pharmaceutical companies shifting away from this field. Additionally, antifungal resistance is increasing and hampering effective treatment (13). As a result, there has never been a greater need for bold and innovative approaches to discovering broad new molecular antifungal targets and their inhibitors.

To meet this need, we propose a new class of antifungals targeting sterylglucosidase (SGL), an enzyme present in fungi but not in human cells. SGL participates in the metabolism of sterylglucosides (SGs), of which ergosterol 3β-d-glucoside (ErgGlc) is the major species in fungi. ErgGlc synthesis involves a sterol glucosyltransferase that adds a single glucose moiety to the 3β-hydroxy group of ergosterol, whereas SGL hydrolyzes ErgGlc to glucose and ergosterol (14).

In the past, our group has been focused on SGL enzymes, especially Cryptococcus neoformans (*Cn*) Sgl1 and *Af* SglA, and how interfering with these enzymes by deletion or pharmacological inhibition to modulate ErgGlc can be used as a novel tool for vaccine and antifungal development. In previous studies, we showed that the genetic ablation of SGL renders the fungi nonpathogenic and that vaccination with a mutant strain lacking this enzyme prevents secondary infections in murine models of cryptococcosis and aspergillosis, which suggests that the accumulation of ErgGlc is involved in the development of protective immunity (15, 16).

In addition, we have determined crystal structures of *Cn* Sgl1 alone and with specific inhibitors (17). In fact, we found that by inhibiting *Cn* Sgl1 with specific inhibitors, we can reproduce the phenotype of ErgGlc accumulation and virulence impairment seen in wild-type cells, preventing brain dissemination in the murine model of cryptococcosis. Thus, we wondered whether this phenomenon would be limited to Cn or would be applicable to other fungi, such as *Af*.

Here, we used high-throughput screening to identify two selective small-molecule SglA inhibitors, which we refer to as hit B and hit C. These compounds induce significant accumulation of ErgGlc *in vivo* and phenocopy the filamentation defect of the *Af* Δ*sgla* mutant. Hits B and C display efficacy in a mouse model of pulmonary aspergillosis and significantly increase the survival of animals. We initiated structure-activity relationship studies, identified 7 derivatives of hit B (B1 to B7), and found that derivative B7 performed better than hit B *in vitro* and in the animal model of aspergillosis. We also determined the crystal structure of SglA and used computational approaches to model the interactions of SglA with its inhibitors to guide future medicinal chemistry optimization.

## RESULTS

### SglA characterization.

**FIG 1:**
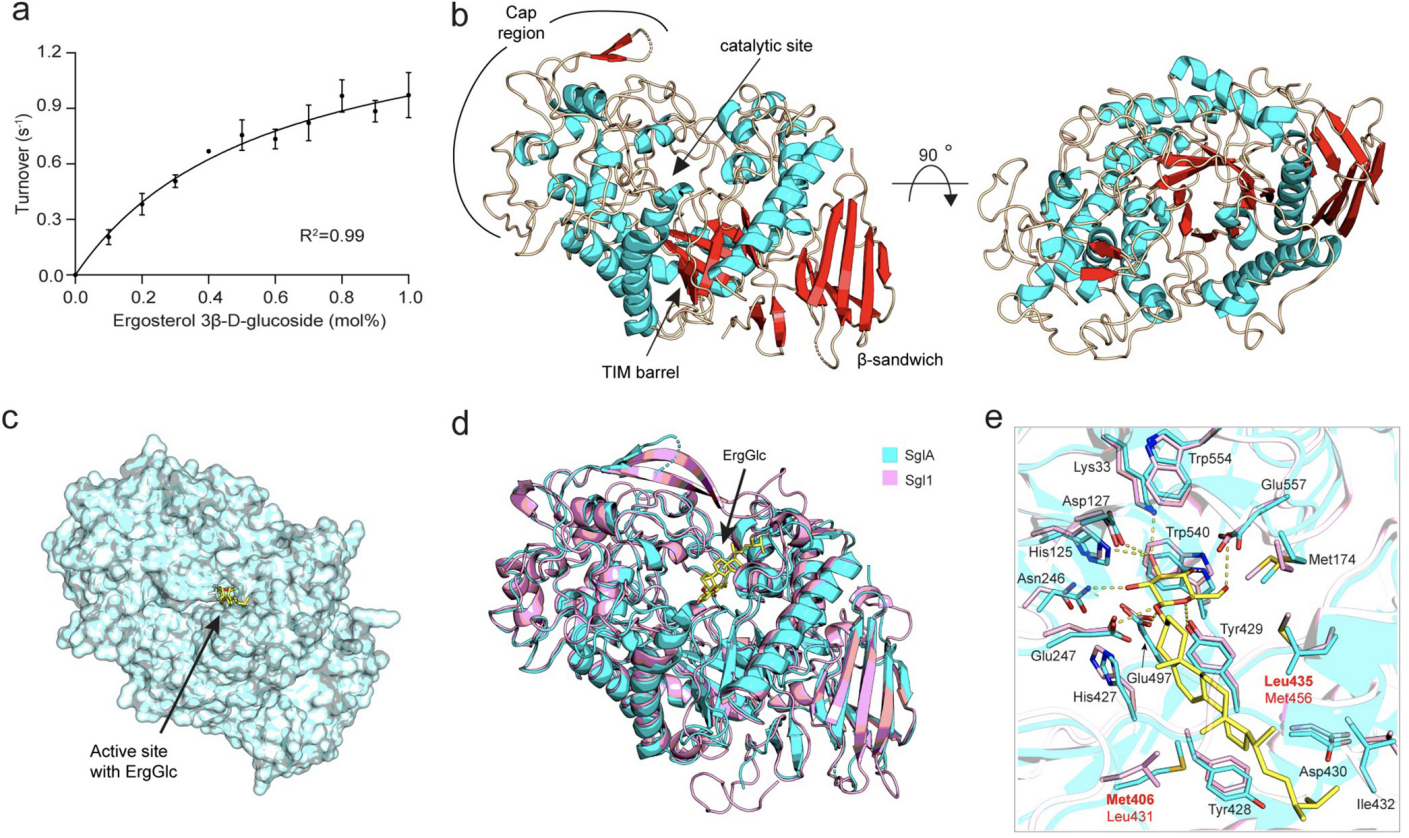
SglA characterization. (a) Kinetic analysis of ErgGlc hydrolysis by SglA (0.11 pmol, 20 min). Data are means and SD from 2 independent experiments. (b) Overall structure of SglA (PDB code: 8EXD). (c) Surface representation of SglA. (d) SglA and Sgl1 (PDB code 7LPQ) structures superimposed. Ergosterol 3β-d-glucoside (ErgGlc) docked into the Sgl1 active site is shown in yellow. (e) Active site of SglA (cyan) superimposed on Sgl1 (light pink) revealed high similarity. SglA residues are labeled. SglA contains identical residues for glucose binding (yellow dashes). SglA Met406 and Leu435 are seen on opposite sides in Sgl1 (Leu431 and Met456) in the ergosterol binding site.

Full-length A. fumigatus SglA was produced in Escherichia coli and purified using affinity and gel filtration chromatographies. The pure protein was used for enzymatic assays and structural determination. To enzymatically characterize SglA, we assessed activity toward ErgGlc that results in the conversion of ErgGlc to ergosterol and glucose (Fig. 1a). The assay was performed under conditions similar to those described previously for *Cn* Sgl1 (17) and quantified the product, ergosterol, by high-performance liquid chromatography (HPLC) (see Fig. S1a to d in the supplemental material). In these experiments, the bulk concentrations of ErgGlc were held constant and the molar ratios of ErgGlc to Triton X-100 were varied to achieve the indicated surface concentrations (expressed as moles percent). SglA readily hydrolyzed ErgGlc with a *K_m_* of 0.57 ± 0.09 mol% in Triton X-100 mixed micelles and a *k*_cat_ of 0.73 ± 0.04 s^−1^ (Fig. 1a).

10.1128/mbio.00339-23.1 FIG S1 Kinetic of ergosterol 3β-d-glucoside (ErgGlc) hydrolysis by SglA. (a) ErgGlc hydrolysis by SglA yields ergosterol and glucose. (b) System linearity of ergosterol detection at 282 nm by UV absorption after HPLC separation. (c) Enzyme concentration linearity of reactions using 0.8 mol% ErgGlc in Triton X-100 mixed micelles with a 20-min reaction time at 37°C. (d) Time linearity of reactions using 0.8 mol% ErgGlc and 0.11 pmol of SglA at 37°C. Download FIG S1, PDF file, 0.4 MB. Copyright © 2023 Pereira de Sa et al. 2023 Pereira de Sa et al. https://creativecommons.org/licenses/by/4.0/ This content is distributed under the terms of the Creative Commons Attribution 4.0 International license.

Moreover, the full-length protein yielded crystals that diffracted to a 3.8-Å resolution (Table 1). Molecular replacement with the *Cn* Sgl1 model was used for solving the SglA structure. The general architecture of SglA was similar to that of Sgl1, with two domains, comprising a catalytic domain with a central TIM (triose-phosphate isomerase) barrel and a C-terminal β-sandwich domain (Fig. 1b and c). SglA also contains a large catalytic domain that forms a cap-like region above the TIM barrel, creating an enclosed Y-shaped cavity, which differentiates *Af* SglA and *Cn* Sgl1 from the bacterial and human homologs that display glucosylceramidase activity (18, 19). These observations reinforce the idea that the unique structures of the sterylglucosidase enzymes are important for substrate specificity.

**TABLE 1:** Data collection and refinement statistics

Statistic	Value for SglA^*a*^
Data collection	
Space group	P 1 21 1
Cell dimensions	
*a*, *b*, *c* (Å)	107.731, 112.792, 139.102
α, β, γ (°)	90, 102.3, 90
Wavelength	1.033
Resolution range	58.21–3.8 (3.936–3.8)
Total reflections	224,683 (22,427)
Multiplicity	7.0 (7.1)
Completeness (%)	98.91 (99.43)
Mean *I*/σ(*I*)	7.72 (1.81)
Wilson B factor	148.32
*R*_merge_	0.1223 (0.8717)
*R*_meas_	0.1322 (0.9415)
*R*_pim_	0.04973 (0.352)
CC_1/2_	0.994 (0.86)
CC*	0.999 (0.962)
Refinement	
Reflections used in refinement	31,957 (3,167)
*R*_work_/*R*_free_	0.2840 (0.3491)/0.3040 (0.3735)
CC_work_/CC_free_	0.882 (0.759)/0.887 (0.726)
No. of nonhydrogen atoms	21,118
Macromolecules	21,118
Ligands	0
Solvent	0
Protein residues	2,607
RMS (bonds)	0.002
RMS (angles)	0.60

a Values in parentheses are for the highest resolution shell.

ErgGlc has not yet been successfully cocrystallized with SglA or Sgl1. However, superimposing the Sgl1 structure containing a docked ErgGlc onto SglA allowed some predictions. The surrounding active-site residues in the glucose binding site are present in the same position as Sgl1 and contain the same capping residues, including a bulky Trp residue that creates a wall to sterically hinder substrates with more than one sugar from binding (Fig. 1d and e). Interestingly, there are slight differences in the composition of active site residues in the sterol binding site, where there is an exchange in positions between methionine and leucine on opposite sides of the pocket (Fig. 1e). We have seen previously in *Cn* Sgl1 protein that the buried active site pocket assumed a Y shape, where there was only one narrow entrance at one of the Y arms (17). However, in SglA, there are minor structural differences with *Cn* Sgl1 that leave a wider opening to the pocket (Fig. S2a and b).

10.1128/mbio.00339-23.2 FIG S2 SglA active-site entrance. (a) The SglA missing loop (red) was modeled within the SglA X-ray structure (gray). The position of this modeled loop does not block the active site, leaving the active site solvent exposed in SglA. (b) Surface model highlighting (cyan dash) the solvent-exposed opening for ErgGlc (yellow) to access the active site pocket. Download FIG S2, PDF file, 1.7 MB. Copyright © 2023 Pereira de Sa et al. 2023 Pereira de Sa et al. https://creativecommons.org/licenses/by/4.0/ This content is distributed under the terms of the Creative Commons Attribution 4.0 International license.

### SglA inhibitors.

**FIG 2:**
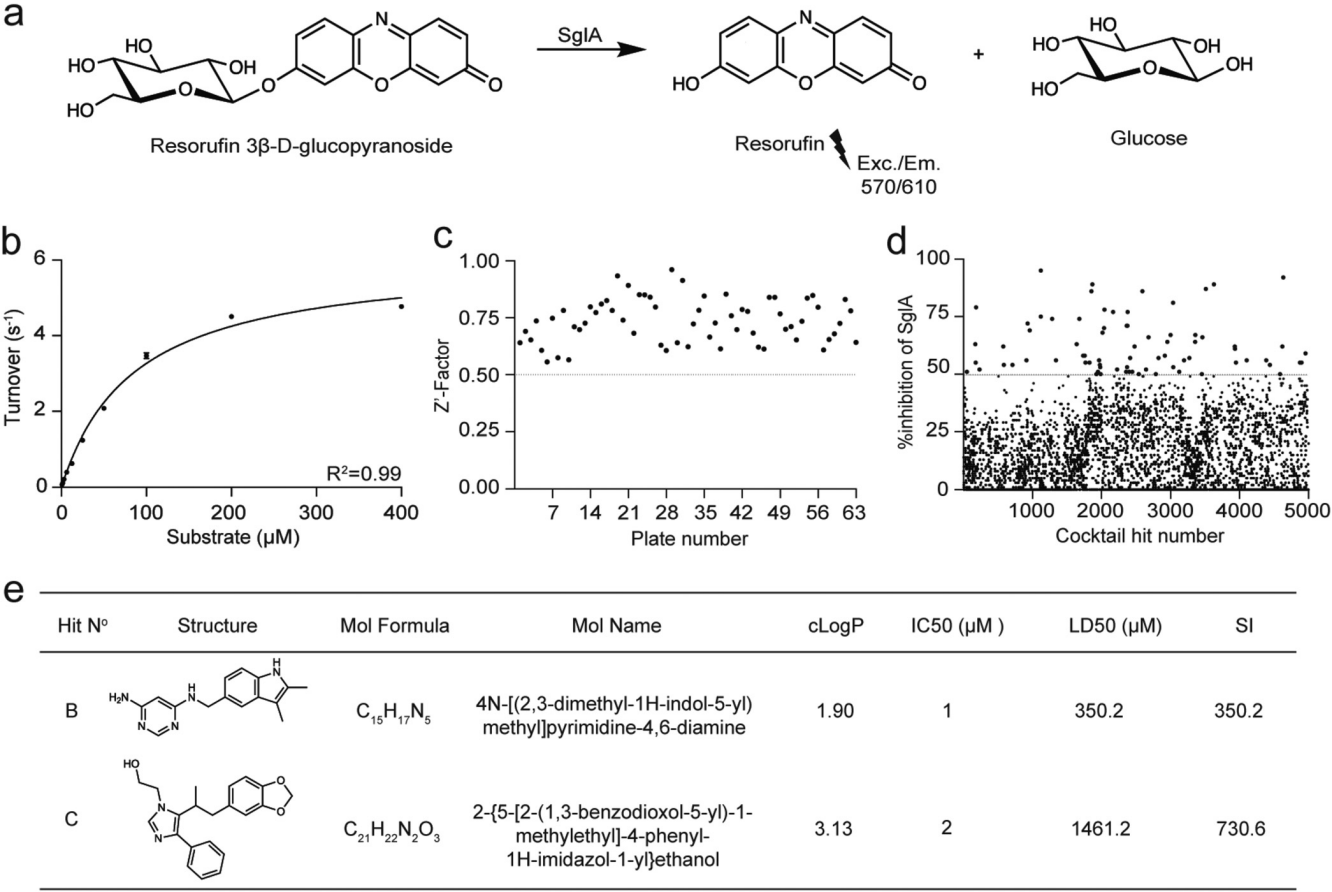
An HTS assay revealed two potent SglA inhibitors with low toxicity. (a) Resorufin 3β-d-glucopyranoside hydrolysis by SglA releases the fluorescent product resorufin and glucose. (b) Kinetic analysis of resorufin 3β-d-glucopyranoside hydrolysis by SglA (0.11 pmol, 20 min). Data are means and SD from 2 independent experiments. (c) Z′ factor graph of 63 cocktail plates. (d) Percentage of inhibition of all 5,000 cocktail hits. Hits that passed the 50% inhibition cutoff are shown in red. (e) Top two hits selected for validation. Hits B and C presented good solubility, good IC_50_s, and low toxicity to human A549 cells (LD_50_ > 350 μM), resulting in an SI (LD_50_/IC_50_) higher than 350.

As A. fumigatus SglA is a promising therapeutic target for the treatment of aspergillosis, we sought to identify small-molecule inhibitors of SglA using a high-throughput screening (HTS) tiered approach (Table S1). The artificial substrate resorufin 3β-d-glucopyranoside (ResGlp) was used, since after hydrolysis it releases the fluorescent molecule resorufin (Fig. 2a). This substrate has been successfully employed in HTS for Sgl1 and other glucosidases (17, 20). After determining the *K_m_* and *k*_cat_ for ResGlp (Fig. 2b; Fig. S3a to h) and optimizing the Z′ factor of our HTS assay (Fig. 2c), we screened 50,000 compounds to identify competitive inhibitors of SglA.

10.1128/mbio.00339-23.3 FIG S3 High-throughput screening assay parameters using resorufin 3β-d-glucopyranoside as a substrate. (a) Detection linearity of the system. (b) Temperature evaluation of SglA activity. (c) Reaction dependence on pH. (d) Enzyme concentration linearity. (e) Time linearity until 40 min. (f) DMSO tolerance. (g) Ten substrate concentrations tested with 0.23 pmol of SglA until 20 min. (h) Kinetic parameters of Sgl1 reaction with resorufin 3β-d-glucopyranoside. Download FIG S3, PDF file, 0.09 MB. Copyright © 2023 Pereira de Sa et al. 2023 Pereira de Sa et al. https://creativecommons.org/licenses/by/4.0/ This content is distributed under the terms of the Creative Commons Attribution 4.0 International license.

10.1128/mbio.00339-23.5 TABLE S1 Small-molecule screening data. Download Table S1, PDF file, 0.05 MB. Copyright © 2023 Pereira de Sa et al. 2023 Pereira de Sa et al. https://creativecommons.org/licenses/by/4.0/ This content is distributed under the terms of the Creative Commons Attribution 4.0 International license.

A cocktail of 10 compounds per well was screened at 5 μM each, and 73 cocktail hits with inhibition equal to or higher than 50% were selected for individual testing (Fig. 2d). From 730 single compounds tested in the concentration range of 0.25 to 4 μM, we identified 20 hits with 50% inhibitory concentrations (IC_50_s) varying from 0.5 to 4 μM (Table S2). Next, we selected compounds with IC_50_s of <2 μM for toxicity evaluation against the mammalian cell line A-549. This indicated that hit B and hit C had low toxicity, with 50% lethal doses (LD_50_) higher than our 200 μM cutoff (Fig. 2e), and yielded selectivity indexes (SIs) of 350 and 730 for hit B and hit C, respectively. Hit B is a pyrimidine derivative with an indole ring attached to it. Hit C is an imidazole derivative with a benzodioxol ring. Both hits B and C have good solubility (cLogP of 1.9 and 3.1, respectively) and IC_50_s of 1 to 2 μM in our primary screen with ResGlp as the substrate (Fig. 2e).

10.1128/mbio.00339-23.6 TABLE S2 Twenty single compound hits identified by HTS. Download Table S2, PDF file, 0.1 MB. Copyright © 2023 Pereira de Sa et al. 2023 Pereira de Sa et al. https://creativecommons.org/licenses/by/4.0/ This content is distributed under the terms of the Creative Commons Attribution 4.0 International license.

### Hit validation.

**FIG 3:**
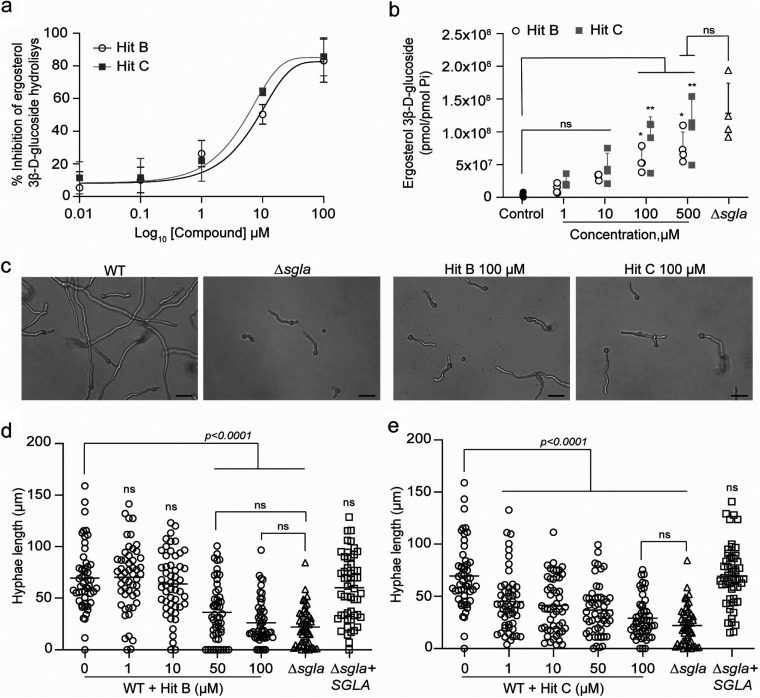
Validation of hits B and C. (a) Dose-response curves against SglA for hits B and C using the natural substrate ErgGlc. Data are means and SD from 2 independent experiments. (b) ErgGlc accumulation in wild-type *Af* after treatment with hit B or C after 48 h of incubation. ErgGlc levels were quantified by LC-MS. Both hits significantly increased ErgGlc levels compared to the control (untreated wild-type *Af*). Statistical analysis by one-way ANOVA with Tukey’s multiple-comparison test. Data are means and SD from 2 independent experiments. (c) Treatment of wild-type *Af* with hit B or C for 12 h recapitulates the phenotype of hyphal elongation delay seen in the *Af* Δ*sgla* mutant. Bar = 20 μm. (d and e) Quantitative analysis of hyphal length of untreated wild-type *Af* (0 μM) and *Af* treated with hit B or C (1, 10, 50, and 100 μM). Both compounds significantly inhibit hyphal elongation in wild-type *Af* similarly to *Af* Δ*sgla*. There was no significant difference observed between the untreated WT and Δ*sgla*+SGLA strains. Statistical analysis by one-way ANOVA with Dunnett’s multiple-comparison test. Data are means and SD from 2 independent experiments. ns, not significant.

The two hits identified in our HTS campaign (hit B and hit C) were submitted to a two-step validation. First, we tested their ability to inhibit the hydrolysis of ErgGlc *in vitro*. Dose-dependent inhibition of ErgGlc hydrolysis by SglA was observed for both compounds, with IC_50_s in the low micromolar range (Fig. 3a).

In the second step, we evaluated the capacity of these SglA inhibitors to promote the accumulation of ErgGlc inside live *Af* wild-type cells. Upon treatment of *Af* wild-type cells for 48 h, we observed a significant and dose-dependent increase in the intracellular concentration of ErgGlc, with the maximal accumulation of ErgGlc at the highest concentration for both compounds (Fig. 3b). Both hits promoted a higher accumulation of ErgGlc at the highest concentrations tested (100 and 500 μM). Hits B and C differed significantly from the control at 100 and 500 μM concentrations after 48 h of treatment. Notably, both hits B and C at 500 μM led to an accumulation of ErgGlc similar to that seen with the *Af* Δ*sgla* strain, where SglA is genetically deleted.

### SglA inhibition promotes a delay in filamentation similar to the mutant *Af* Δ*sgla*.

Fernandes et al. (16) found that the *Af* Δ*sgla* strain presents a delay in the initial stages of filamentation with shorter hyphal length at 37°C after 12 h, which delayed fungal growth compared with the wild-type strain. We sought to use this assay to further validate SglA inhibition, with the expectation that the SglA inhibitors would reproduce this phenotype in the *Af* wild-type strain. Thus, we assessed hyphal length after treatment with hit B and hit C at a range of concentrations between 1 and 100 μM. We observed that hit B significantly delays the germination and hyphal growth of *Af* wild-type conidia at 50 and 100 μM, and at 100 μM, the hyphal length was similar to that in the *Af* Δ*sgla* mutant strain (Fig. 3c and d). Hit C significantly reduced hyphal growth at all concentrations tested (Fig. 3e), with results for 100 μM hit C being similar to those for the *Af* Δ*sgla* strain.

### Hit B and Hit C increase survival in a mouse model of pulmonary aspergillosis.

Encouraged by these results, we assessed the therapeutic potential of SglA inhibition by hits B and C in a well-characterized mouse model of pulmonary aspergillosis. In an initial experiment, male and female CBA/J mice were immunosuppressed with triamcinolone acetonide, infected intranasally with conidia of the *Af* wild-type strain Δku80pyrG^+^, and treated daily by intraperitoneal administration of either 5 or 20 mg/kg/day of hit B, 20 or 60 mg/kg/day of hit C, or vehicle (5% Solutol in phosphate-buffered saline [PBS]).

**FIG 4:**
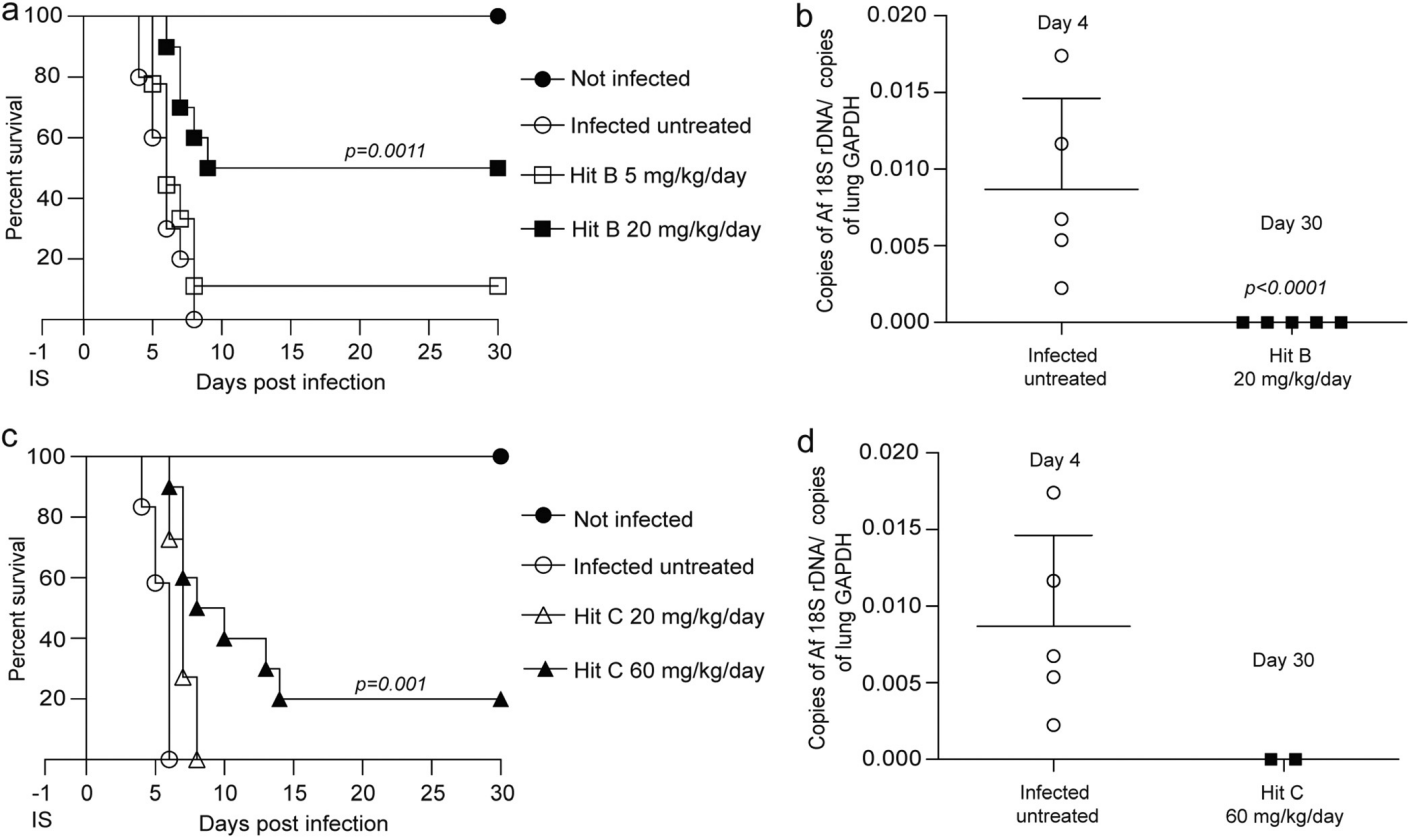
Inhibition of SglA by hit B or C reduces Aspergillus fumigatus virulence in a mouse model of pulmonary aspergillosis. (a) Virulence study showing that 50% of CBA/J mice receiving an intranasal administration of 2.5 × 10^4^ conidia of wild-type *Af* and treated with hit B at 20 mg/kg/day intraperitoneally survived the infection, whereas mice that were infected and untreated died within 8 days. *n* = 10 mice/group. (b and d) All mice that survived 30 days after the WT challenge exhibited full recovery from the infection. The lung fungal burden was determined by real-time qPCR based on the 18S rRNA gene of *Af* and an intronic region of the mouse GAPDH gene on day 4 after infection. Statistical analysis by one-way ANOVA with Tukey’s multiple-comparison test. Data are means and SD for 5 mice. (c) Intraperitoneal treatment with hit C significantly prolonged mouse survival in comparison with the control (infected untreated mice). IS, immunosuppression (all mice received a subcutaneous administration of triamcinolone acetonide [100 mg/kg] 1 day prior to infection with wild-type *Af* conidia). Statistical analysis of survival curves was performed by the log-rank (Mantel-Cox) test.

Both compounds significantly reduced the virulence of wild-type *Af*. Treatment with hit B at a dose of 20 mg/kg/day prolonged the survival of 50% of the animals, which fully recovered after 30 days of treatment, with no fungal burden in the lung tissue (Fig. 4a and b). In contrast, hit C significantly prolonged survival at the higher dose of 60 mg/kg/day, but only 20% of animals survived until day 30 postinfection with full lung clearance (Fig. 4c and d). Overall, SglA inhibition displays efficacy in controlling A. fumigatus infection *in vivo*, thus validating SglA as a potential antifungal target.

### SglA inhibitor modeling.

To aid future medicinal chemistry optimization, we used *in silico* analysis to dock hit B and hit C into the SglA active-site pocket, since our efforts to cocrystallize SglA with both hit compounds have so far been unsuccessful. The binding site for these compounds in SglA was defined by overlaying the SglA structure with the high-resolution cocrystal structure of the *Cn* Sgl1-hit 9 complex (PDB code 7LPQ) in UCSF Chimera.

**FIG 5:**
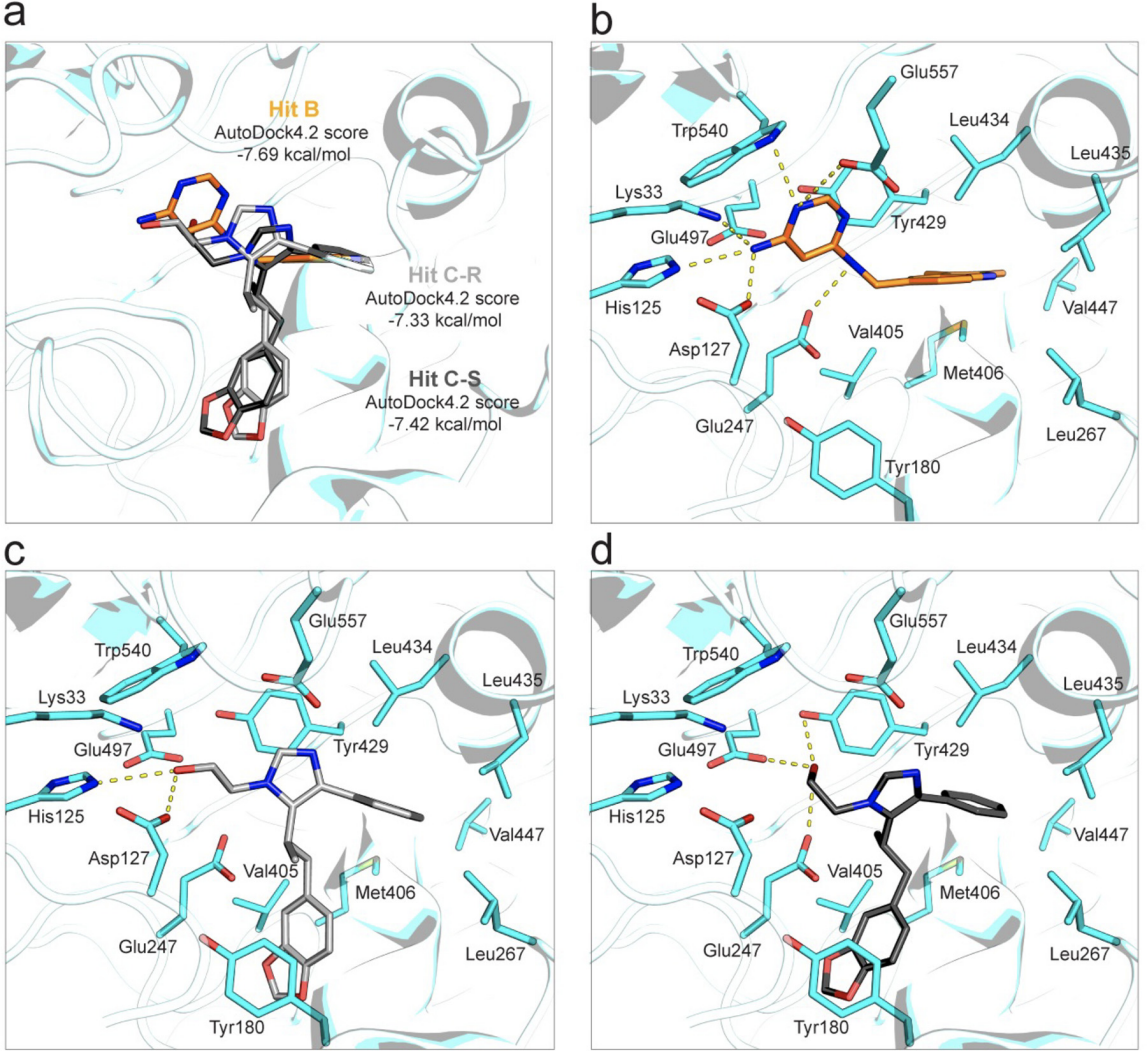
Hits B and C docked in the active site of SglA. (a) Positions of hits B and C at the bottom of the active-site pocket. Hit C would possibly assume two conformations (R and S). AutoDock4.2 docking scores are indicated. (b) Hit B makes hydrogen bond contacts with Glu247, Asp127, His125, Lys33, Trp540, and Glu557 in the glucose binding site. (c) The hit C-R free hydroxyl moiety interacts with Asp127 and His125. (d) The hit C-S conformation would make hydrogen bond contacts with Glu247, Glu497, and Tyr429.

The hit B pyrimidine ring bound within the active site and formed a series of polar interactions near the catalytic site while the indole ring occupied the space close to the hydrophobic portion of the pocket (Fig. 5a). The pyrimidine ring of hit B formed hydrogen bond interactions with the active-site residues Glu247, Asp127, His125, Lys33, Trp540, and Glu557 at the sugar-binding site (Fig. 5b).

The hit C imidazole moiety resides in proximity to the hit B indole ring; however, the interpretation of the hit C predictions was not as straightforward as for hit B, since two conformations, R and S, were identified. The main difference between the two conformations attributes in the direction of the hydroxyl group. The hit C (R) conformation has its hydroxyl group forming key interactions with His125 and Asp127 (Fig. 5c), whereas hit C (S) would make a hydrogen bond contact with Glu247, Glu497, and Tyr429 (Fig. 5d). Cocrystallization is still needed for the confirmation of the hit B and C binding poses.

### Hit B derivatives.

**FIG 6:**
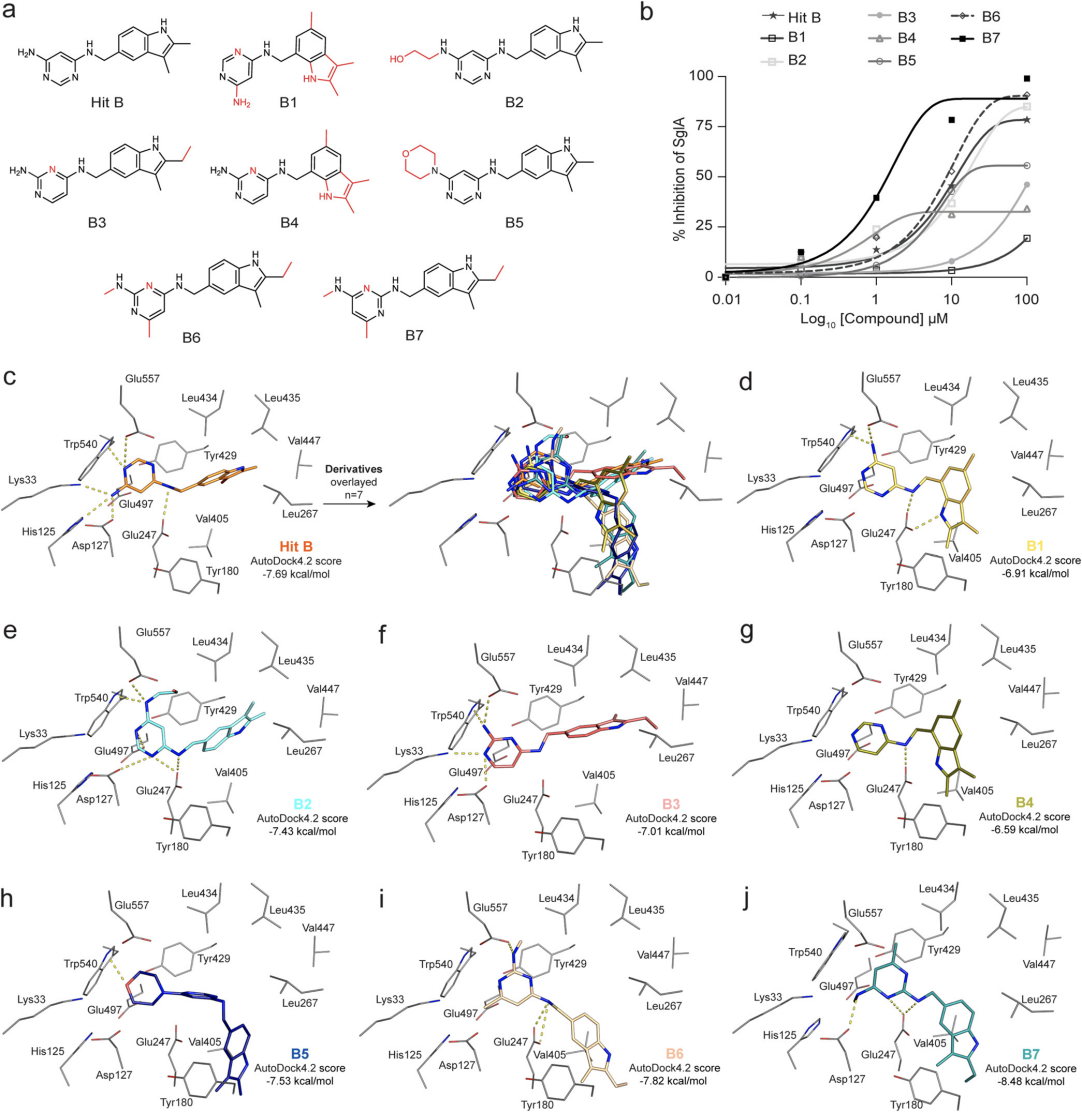
Structure activity relationship studies identify a more potent hit B derivative. (a) Chemical structures of the seven hit B derivatives highlighting the chemical modifications (red). (b) Dose-response curve of hit B and its derivative compounds tested against SglA using ErgGlc as a substrate. (c to j) 3D poses and AutoDock4.2 docking scores of hit B derivatives in the SglA active site. (c) Hit B. (d) Compound B1. (e) Compound B2. (f) Compound B3. (g) Compound B4. (h) Compound B5. (i) Compound B6. (j) Compound B7. The closest key residues involved in H bonding (yellow dashes) are highlighted.

The corroboration of the *in vitro* and *in vivo* results reinforced the potential of hit B and hit C as antifungals. Therefore, we looked in the ChemBridge library for compound derivatives of hit B and hit C to conduct a limited structure-activity relationship study. Whereas we found no derivatives for hit C, we found seven compounds with chemical features similar to those of hit B, with cLogP values ranging from 2.36 to 3.57 (Table S3). The seven hit B derivatives are referred to as B1 to B7. All hit B derivatives conserved the pyrimidine and indole moieties and changed mostly the position of the amines throughout the pyrimidine ring or/and modified the methylamine group attached to the hit B structure (Fig. 6a). In the indole ring, other modifications are also present (Fig. 6a).

10.1128/mbio.00339-23.7 TABLE S3 Hit B derivatives. Download Table S3, PDF file, 0.07 MB. Copyright © 2023 Pereira de Sa et al. 2023 Pereira de Sa et al. https://creativecommons.org/licenses/by/4.0/ This content is distributed under the terms of the Creative Commons Attribution 4.0 International license.

We assessed the potential inhibition of SglA enzymatic activity toward ErgGlc by the seven derivatives at concentrations of 0.1, 1, 10, and 100 μM. The results showed that the chemical modifications in derivatives B1, B3, and B4 reduced the capacity to inhibit SglA, resulting in IC_50_s higher than 100 μM, while hit B exhibited an IC_50_ of 10 μM with ErgGlc as the substrate (Fig. 6b). Derivatives B2 and B5 exhibited IC_50_s of 15 μM, and B6 had an IC_50_ of 9 μM, which are all similar to that of hit B. Interestingly, the derivative B7 performed much better than hit B, with an IC_50_ of 3 μM.

Thus, we docked all seven derivatives following the protocol used for hit B (Fig. 6c to j). The compound’s pose and interactions corroborated the *in vitro* results, showing that the derivative B7 is capable of making more hydrogen bond contacts in the SglA active site than hit B or the other derivatives, with an improvement in the docking fitness score from −7.69 kcal/mol (hit B) to −8.48 kcal/mol (B7) (Fig. 6c and j; Fig. S4). Interestingly, unlike hit B, B7 also makes noncovalent interactions with Tyr429 by direct stacking of aromatic rings of the B7 pyrimidine ring with Tyr429. Although the B7 rings are too far away from Tyr180 to be involved in π-π stacking, these rings are predicted to also make strong Van der Waals interactions with Tyr180. Taking these results together, we predict that these interactions are responsible for the higher affinity of B7 for the SglA active site.

10.1128/mbio.00339-23.4 FIG S4 Docking poses of hit B and B7 at the binding site of SglA (hydrophobicity surface view). The pyrimidine ring on hit B and B7 has oriented toward the narrow polar region of the binding pocket of SglA, while the methyl-substituted indole ring of hit B points toward the entrance of the binding pocket surrounded mainly by hydrophobic residues mainly. As opposed to hit B, the indole ring of B7 points toward the broader region of the binding site of SglA surrounded by more hydrophilic residues while keeping the ethyl group of the indole ring in closer proximity to hydrophobic residues, making B7 stable inside the binding site. Download FIG S4, PDF file, 4.2 MB. Copyright © 2023 Pereira de Sa et al. 2023 Pereira de Sa et al. https://creativecommons.org/licenses/by/4.0/ This content is distributed under the terms of the Creative Commons Attribution 4.0 International license.

### B7 validation *in vitro* and *in vivo*.

**FIG 7:**
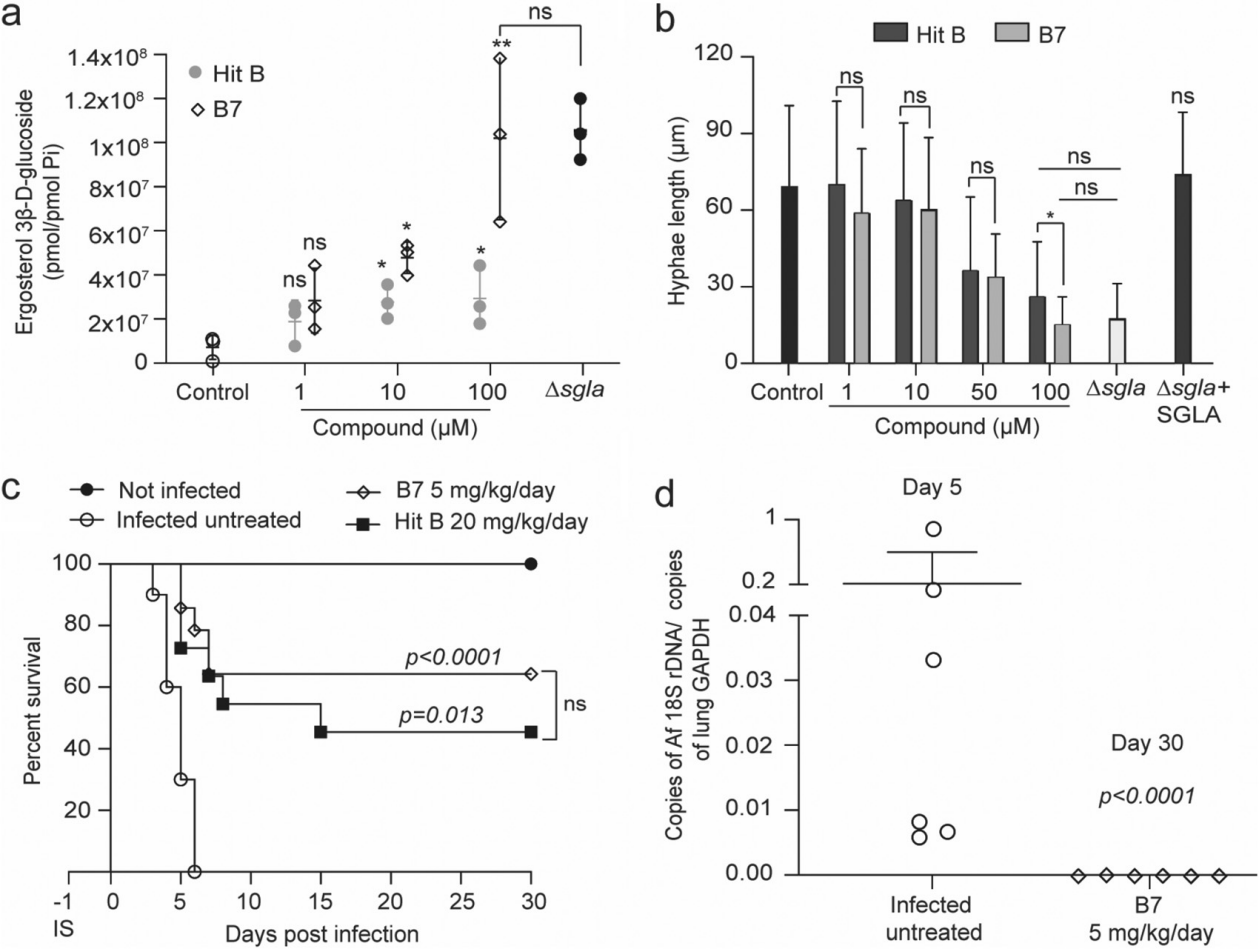
The more potent derivative B7 has better performance *in vitro* and *in vivo* than the parent hit B compound. (a) B7 promotes significant accumulation of ergosterol 3β-d-glucoside (ErgGlc) in wild-type *Af* in comparison to the control (untreated wild-type *Af*). At 100 μM, B7 leads to a significantly higher accumulation of ErgGlc than hit B, at a level similar to that seen with the Δ*sgla* mutant. Statistical analysis by one-way ANOVA with Dunnett’s multiple-comparison test. Data are means and SD from 2 independent experiments. (b) B7 treatment at 50 and 100 μM during 12 h culminates in shorter hyphae than hit B. (c) Sixty percent of CBA/J mice receiving an intranasal administration of 2.5 × 10^4^ conidia of wild-type *Af* and treated intraperitoneally with B7 at 5 mg/kg/day survived the infection, whereas infected untreated mice died within 6 days (*n* = 10 mice/group). The survival rate was similar to that seen with hit B treatment at 20 mg/kg/day (*P* > 0.05). Statistical analysis was performed by a log-rank (Mantel-Cox) test. (d) The animals that survived 30 days after the wild-type challenge presented full recovery from the infection. The lung fungal burden was performed by qPCR determination of the concentration of fungal 18S DNA compared to the concentration of GAPDH from mouse lungs on day 4 after infection. Statistical analysis by one-way ANOVA with Tukey’s multiple-comparison test. Data are means and SD for 6 lungs. ns, not significant; *, *P* < 0.05; **, *P* < 0.01.

Since B7 showed a better affinity for SglA than hit B, we assessed the ability of this compound to promote the accumulation of ErgGlc and to delay hyphal elongation in the *Af* wild-type strain. B7 treatment for 48 h of wild-type *Af* led to a significantly higher accumulation of ErgGlc than treatment with hit B at 100 μM (Fig. 7a). Moreover, this accumulation at 100 μM is similar to the accumulation observed in the mutant Δ*sgla* strain. In addition, B7 treatment promotes a significant delay in hyphal growth, performing even better than hit B at 100 μM, resulting in hyphal lengths that are similar to those in the Δ*sgla* strain (Fig. 7b). The toxicity of B7 in the mammalian cell line A549 was slightly worse than that of the parent compound, with an LD_50_ of 200 μM, compared to 350 μM for hit B.

### B7 increases survival at a lower dose in a mouse model of pulmonary aspergillosis.

With these promising results, we then tested B7 at 5 mg/kg/day intraperitoneally in the same murine model of aspergillosis, using hit B at 20 mg/kg/day as a control. Once more, we confirmed that B7 performed better than hit B, being able to promote full recovery of 60% of animals infected with the *Af* wild-type strain at the lower dose of 5 mg/kg/day (Fig. 7c and d). These results were similar to the 50% survival rate promoted by hit B, which used a 4-fold higher dose of 20 mg/kg/day. This reinforces the idea that compounds with higher affinity for SglA tend to perform better *in vitro* and also correlates with an improved performance in animals.

## DISCUSSION

Here, we identified specific inhibitors of SglA that cause accumulation of ErgGlc in cells, reproduce the filamentation delay observed in the Δ*sgla* mutant strain, and are efficacious in a mouse model of pulmonary aspergillosis. Furthermore, we identified a derivative that has a higher affinity for SglA and performs even better than the original hits *in vitro* and in animals. This work reveals a new therapeutic strategy for treating aspergillosis and provides an important starting point for medicinal chemistry optimization of more effective SglA inhibitors, which can take advantage of the structural information we obtained for *Af* SglA.

*Af* SglA is the second sterylglucosidase to be characterized, after *Cn* Sgl1. Given the universal conservation of sterylglucosidases in fungi, but not humans, and the fact that both genetic and chemical inhibition display efficacy in mouse models, this presents an opportunity to design broad-spectrum inhibitors for sterylglucosidases in pathogenic fungi. Alternatively, there is also an opportunity to develop specific inhibitors for individual sterylglucosidases, which could be selectively used for different fungal pathogens. The later strategy could take advantage of the minor differences observed at the sterol binding site to enable design of specific inhibitors for SglA or Sgl1. In contrast, the nearly identical active site pocket may enable identification of a single compound that inhibits all fungal sterylglucosidases and could be used indiscriminately as a broad-spectrum antifungal agent.

Based on a recent study from our group (16), it became apparent that SglA is a key virulence factor in A. fumigatus, since genetic deletion of SglA caused a significant delay in hyphal differentiation and the production of a dense polysaccharide-rich extracellular matrix, which can result in impairment of adhesion and biofilm formation. Compromising these abilities would slow the progress of the infection once the conidia are inhaled by a host. At the same time, the absence of SglA function in the fungal cell led to accumulation of ErgGlc, which is essential for inducing a protective immune response by the host, even if it is immunocompromised. In fact, Fernandes et al. (16) also demonstrated that similar to C. neoformans, the genetic ablation of SglA in A. fumigatus is nonpathogenic in primary infection in mice. In addition, animals vaccinated with live or heat-killed A. fumigatus Δ*sgla* conidia exhibit complete protection against a subsequent challenge with wild-type A. fumigatus. Consequently, by inhibiting SglA pharmacologically, it may be possible to stimulate an efficient immune response against the primary infection and possibly induce protection against a secondary infection using a pharmacological agent.

We propose that pharmacological inhibition of SglA is a promising therapeutic approach, since our results shows that SglA inhibition by hit B, hit C, and the B7 derivative significantly reduced wild-type *Af* virulence, prolonging survival. Hit C performed better than hit B *in vitro*, although *in vivo* it increased survival only at very high doses (60 mg/kg/day), which could be related to the biophysical and pharmacokinetic properties of the compounds. In fact, hit C is much less soluble than hit B, perhaps limiting its absorption upon intraperitoneal injection. Another venue yet to be explored to improve the survival rate is the interaction of SglA inhibitors with other antifungals *in vivo*, especially azole drugs, since in the absence of sterylglucosidase activity when the biosynthetic pathway of ergosterol is inhibited by azoles, there would be a complete lack of ergosterol production by the fungal cell that could dramatically affect cell functioning. In addition, the delay in the hyphal growth retarding the infection progress could positively impact the efficiency of other antifungal therapies.

Our finding using derivative B7, which has a higher affinity for SglA, suggests that the optimization of inhibitor potency and pharmacological properties may enhance the drug efficacy to eventually improve survival to 100%, as seen in the *Af* Δ*sgla* mutant (16). Interestingly, hit B and its derivatives are characteristically heterocyclic compounds with pyrimidine and indole constituents, which are of immense chemical and biological significance and present in many natural and synthetic bioactive drug-like molecules (21, 22). From the 20 hits found in our HTS campaign, pyrimidine and indole rings are found in almost half of the compounds (Table S1). Pyrimidine-indole derivatives have been reported to inhibit α-glucosidase and α-amylase (23). Among the nitrogen-containing heterocycles, indole derivative compounds have been reported to have a wide range of biological activities, including antibacterial, antifungal, anticancer, and anti-HIV properties (24–27). However, to our knowledge, there are no inhibitors with chemical structures similar to that of hit B and its derivatives that target other β-glucosidases. Thus, this work is highly innovative.

**FIG 8:**
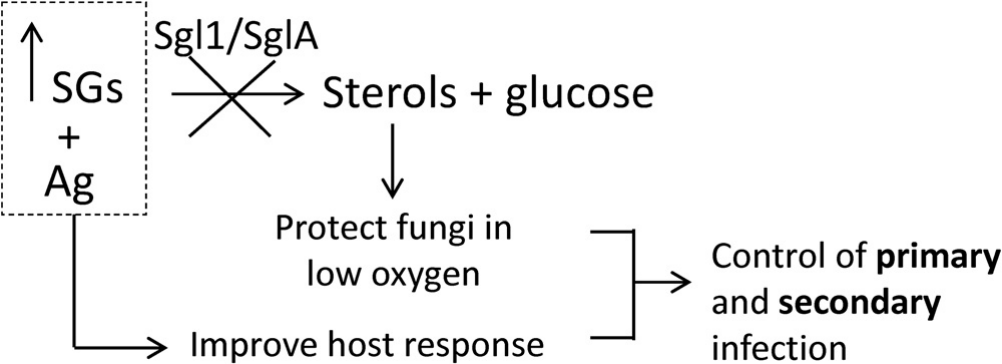
Schematic representation of the effect on targeting/deleting Sgl1/SglA. Deletion of the *SGL1* gene in C. neoformans causes accumulation of SGs, leading to a growth defect at low oxygen levels. Deletion of the *SGLA* gene in A. fumigatus also causes accumulation of SGs, leading to a defect in hyphal elongation. Both of these phenotypes can be recapitulated by targeting Sgl1 or SglA therapeutically.

In our previous studies, we found that C. neoformans Δ*sgl1* and A. fumigatus Δ*sgla* are potent immune stimulators, and for C. neoformans Δ*sgl1*, we showed that this immune activation is controlled by Toll-like receptor 2 (TLR2) on γ/δ T cells (28). Thus, both *Cn* Δ*sgl1* and *Af* Δ*sgla* are exciting vaccine candidates, because they are highly effective in preventing a secondary infection either as live-attenuated or as heat-killed vaccines. Hence, we envision that a drug targeting Sgl1/SglA, and thus increasing SGs, would stimulate a protective immunity which will help in the clearance of the primary infection and potentially in preventing the recurrence of a secondary infection (Fig. 8). This type of treatment could be ideal in patients waiting for transplants (who are susceptible to aspergillosis) and in patients affected with advanced HIV infection (who are susceptible to cryptococcosis), because these fungal vaccines are effective in neutropenia (a condition that favors aspergillosis) and CD4^+^ T cell deficiency (a condition that favors cryptococcosis). Thus, our results may open the avenue to a totally new field of basic research and clinical investigation.

In conclusion, we discovered a new class of antifungal inhibitors targeting a fungal specific enzyme (SglA) that are efficacious *in vitro* and *in vivo* in decreasing the virulence of *Af*, a life-threatening fungus for which a new treatment option is desperately needed. We determined the structure of SglA, predicted the binding poses of these inhibitors through docking analysis, and identified a more efficacious derivative with a limited SAR study. This presents several exciting avenues for the research and development of a new class of antifungal agents targeting sterylglucosidases.

## MATERIALS AND METHODS

### Chemical compounds.

A 50,000-compound DIVERSet-CL library and individual hits were purchased from ChemBridge (San Diego, CA). Resynthesized hits B and C were purchased from the ICB&DD Chemistry Laboratory (Stony Brook, NY). Hit B derivatives and B7 compound that had undergone large-scale resynthesis were purchased from ChemBridge.

### Plasmid.

The Aspergillus fumigatus SglA gene was synthesized, codon optimized for expression in E. coli, and inserted into a pET28a+ plasmid by BioBasic (Canada).

### A. fumigatus SglA overexpression and purification.

The A. fumigatus SglA plasmid was transformed into BL21(DE3) RIPL cells (Agilent Technologies) for protein overexpression according to methods previously described by Pereira de Sa et al. (17). Cells were grown at 37°C in Terrific Broth to an optical density at 600 nm (OD_600_) of 1.5 and then cooled at 10°C for 2 h. Next, protein expression was induced with 100 μM isopropyl β-d-1-thiogalactopyranoside (IPTG) at 15°C overnight before harvesting. Cell pellets from 1 L culture were resuspended in a lysis buffer composed of 50 mM Tris (pH 7.5), 500 mM NaCl, 60 mM imidazole, 5% glycerol, 1% Triton X-100, and 2 mM β-mercaptoethanol (βME) and then lysed by sonication at an amplitude of 85 with cycles of 2 s over 1.5 min. This procedure was repeated 5 times, and the resulting cell lysates were centrifuged at 48,380 × *g*. Thereafter, A. fumigatus SglA was purified using a HisTrap FF column and eluted in buffer with an increased imidazole concentration of 300 mM in 50 mM sodium citrate buffer (pH 5) with 500 mM NaCl, 5% glycerol, and 2 mM βME. The resulting fractions were supplemented with 8 mM βME and 10 mM dithiothreitol (DTT), and then the protein was applied to a Superdex 26/60 HiLoad 200 column (GE Healthcare) equilibrated with 50 mM sodium citrate buffer (pH 5), 150 mM NaCl, 10 mM βME, and 2 mM DTT. Purified protein was concentrated to 10 mg/mL, flash frozen, and stored at −80°C.

### Crystallization and data collection.

The crystallization process was performed using hanging drop vapor diffusion with a well solution of 20% polyethylene glycol (PEG) 3350, 0.2 M magnesium acetate (pH 4.5) at room temperature. and 1.5 μL of *Af* SglA at 6.5 mg/mL was added to an equal volume of the well solution. Microseeds derived from previous crystal drops under the same conditions were diluted 1:100, and 0.2 μL was added per drop. The crystals obtained were frozen in a cryoprotectant solution containing the same components as the drop solution with addition of 40% glycerol. Diffraction data were collected at the Advanced Photon Source GM CAT 23ID-B beamline at Argonne National Laboratory in 15° wedges. All data were processed using xia2 DIALS in CCP4 (29–31).

### Structure determination and refinement.

The *Af* SglA phasing procedure was carried out in Phenix using Phaser for molecular replacement with the 2.13-Å C. neoformans Sgl1 (PDB code 7LPQ) and Autobuild (32–34). Additional model building in Coot (35) and refinement in Phenix produced the final model (Table 1) (PDB code 8EXD).

### Kinetic evaluation of ergosterol 3β-d-glucoside.

A 50-μL volume of wild-type *Af* SglA containing 10 ng of protein (0.11 pmol) and 50 μL of mixed micelles of lipid and Triton X-100 in 50 mM sodium acetate buffer (pH 5.5) with 150 mM NaCl, 5 mM βME, and 5 mM DTT were mixed and incubated at 37°C for 20 min (ErgGlc; Avanti Polar Lipids). After the incubation period, each reaction was quenched with a 2:1 chloroform-methanol solution, and the organic phase was collected and dried. Then, the lipid content was resuspended in 50 mL methanol and analized by HPLC using Agilent 1260 Infinity II (Agilent Technologies). Total ErgGlc and ergosterol were detected at 282 nm on a C_8_ column with a flow rate of 0.5 mL/min in methanol-water (90:10) buffered with 1 mM ammonium formate and 0.2% formic acid.

### Resorufin 3β-d-glucopyranoside enzymatic assay.

Resorufin 3β-d-glucopyranoside (ResGlp; Sigma-Aldrich) enzyme kinetic assays were performed according to methods described by Pereira de Sa et al. (17). Reaction buffer contained 50 mM citric acid, 176 mM K_2_HPO_4_ (pH 6), 0.01% Tween 20, and 10 mM sodium taurocholate. The reaction was carried out in a black 96-well plate using 0.23 pmol of SglA with 100 μM ResGlp substrate. 10 μL/well of ResGlp was added to each well and the reaction was initiated by adding 20 μL enzyme (0.23 pmol). After incubation at 37°C for 30 min, the fluorescence was read at an excitation of 570 (±10) nm and an emission of 610 (±10) nm on a VersaMax microplate reader from Molecular Devices. A standard curve was prepared with serial dilutions of the free fluorophore, resorufin, in the same volume of assay buffer. All reactions were linear with respect to time and protein concentration, and the pH, temperature, and dimethyl sulfoxide (DMSO) optimal tolerance were verified.

### HTS for SglA inhibitors.

A ChemBridge DIVERSet-CL library (ChemBridge, San Diego, CA) containing 50,000 compounds was screened to identify small molecules that inhibit *Af* SglA. The library was prepared in a 96-well plate format containing a cocktail of 10 compounds per well at 1 mM each in 100% DMSO. The cocktail plates were first diluted to 100 μM each (1:10 dilution in Dulbecco’s phosphate-buffered saline [DPBS]), resulting in 10% DMSO. In order to screen the compound cocktails at 1 μM in 1% DMSO in a final reaction volume of 30 μL, an aliquot of 3 μL was added to 17 μL of buffer containing 0.23 pmol of enzyme and the reaction was started by adding 10 μL of 100 μM ResGlp. A negative control without enzyme and positive controls with and without DMSO were prepared. The plates were incubated at 37°C for 30 min. The Z′ factor was used for the assessment of the efficiency of the HTS assay for each plate. Values greater than 0.5 are considered indicators of excellent screening assay quality (36). Compound cocktails from plates with Z′ factors higher than 0.5 and showing >50% inhibition compared to the control well (1% DMSO but no drug) were selected for tests, with each compound used in serial dilutions from 0.25 to 4 μM.

### Toxicity evaluation of hits.

The single compounds with IC_50_s below 2 μM were selected for toxicity evaluation against the human lung epithelial cancer cell line A549. The cells were maintained in Dulbecco’s modified Eagle medium (DMEM) containing 10% fetal bovine serum (FBS) and 1% penicillin-streptomycin. At passage 11, 10^5^ cells were transferred into each well of 96-well plates and cultured for 18 to 24 h to allow the cells to adhere to the well surface. Next, the medium was removed, and fresh medium containing the selected compounds at concentration range of 1 to 512 μM was added to the wells. Controls with equivalent serial concentrations of DMSO were also evaluated and compared with a control without DMSO. The plate was incubated at 37°C with 5% CO_2_. After 24 h, the supernatant was removed and 50 μL of 5 mg/mL 3-(4,5-dimethylthiazol-2-yl)-2,5-diphenyltetrazoliumbromide (MTT) solution in PBS was added to each well. The plates were incubated for an additional 4 h, and the formazan crystals formed inside the cell were dissolved by adding 50 μL DMSO. Next, the absorbance was measured at 570 nm, and the calculated LD_50_ was divided by the IC_50_ of each hit in order to determine the SI of each compound. Compounds with SIs higher than 200 were considered for further validations.

### Ergosterol 3-β-d-glucoside accumulation.

Wild-type *Af* ΔKu80pyrG1a and the mutant Δ*sgla* strain were cultivated in yeast extract-glucose medium (YAG) (2% [wt/vol] glucose, 0.5% [wt/vol] yeast extract, 1× trace elements, 1× amino acid solution, 2% [wt/vol] bacteriological agar) for 48 h at 37°C. High-nitrate salts, trace elements, and amino acid solutions were prepared as previously described (37). The medium was supplemented with 1.2 g/L of uracil and uridine (UU), generating YAG+UU medium when the strain ΔKu80pyrG1a was used. After that, the a conidial suspension in water was obtained, and a pellet with 1 × 10^7^ conidia was treated in minimal medium broth pH 6.5 (MM or MM+UU) (1% [wt/vol] glucose, 1× high-nitrate salts, 1× trace elements) for 48 h at 37°C under agitation, with the hits selected, according to the toxicity criteria, at various concentrations. A previously described MIC assay was performed in accordance with the guidelines in the CLSI document M38-A2 (38), to determine the MIC and select a concentration range that does not affect mold growth. After that, the resultant hyphal pellet was used for lipid extraction. Then, the total lipid was extracted as described by Singh et al. (39). The dried samples were resuspended in 2:1 chloroform-methanol for liquid chromatography-mass spectrometry (LC-MS) analysis. A standard ErgGlc from Avanti Polar Lipids was used as a control for the calibration curve. Data were normalized to the total inorganic phosphate content in the sample.

### Filamentation assay.

To evaluate hyphal growth, conidial suspensions of the *Af* wild-type strain ΔKu80pyrG1a and the Δ*sgla* and Δ*sgla*+*SGLA* strains were obtained as described above. Then, the assay conditions were performed as previously described by Fernandes et al. (16). For that, 1 × 10^5^ conidia of strain ΔKu80pyrG1a were inoculated onto a glass-bottom dish containing 2 mL of MM or MM+UU with and without the experimental compounds in various concentrations and grown at 37°C for 10 to 12 h. MM broth without the compounds were used for the mutant and the reconstituted strains. The living hyphae growing attached to the glass were analyzed by differential inference contrast (DIC) microscopy using a Zeiss Observer D.1 microscope, and the hyphal length was measured using ImageJ software.

### *In vivo* assays.

Male and female CBA/J mice 5 to 6 weeks old were purchased from Envigo and allowed 1 week to acclimate upon arrival. One day prior to infection, the animals were immunosuppressed with 100 mg/kg of the corticosteroid triamcinolone acetonide (Alfa Aesar) subcutaneously as previously described (16). On the day of the infection, the animals were initially anesthetized with a ketamine-xylazine solution (95 mg of ketamine and 5 mg of xylazine per kg of body weight) intraperitoneally and then infected with 2.5 × 10^4^ *Af* wild-type ΔKu80pyrG+ conidia in 20 μL of PBS intranasally. One hour after the procedure, intraperitoneal treatment with hit B (5 and 20 mg/kg/day) and hit C (20 and 60 mg/kg/day) was started and continued until 30 days postinfection. Mice that survived until day 30 were euthanized by CO_2_ inhalation and used for organ fungal burden determinations. A similar procedure was performed for derivative B7 at a dose of 5 mg/kg/day.

### Lung fungal burden quantification by qPCR.

Lung fungal burden was assessed by the determination of the amplification of fungal 18S DNA by qPCR. The lungs of euthanized mice were aseptically removed and immediately frozen in liquid nitrogen. Genomic DNA extraction was carried out as previously described by Malavazi and Goldman (40) with modifications. Previous to the genomic DNA extraction, the lungs were lyophilized for 2 to 3 days, ground using 2-mm glass beads, resuspended in 1 mL of lysis buffer, and vortexed thoroughly. One milliliter of phenol-chloroform–isoamyl alcohol (25:24:1), stabilized and saturated with 100 mM Tris-EDTA to pH 8.0 (Sigma-Aldrich), was added, and the mixture was vortexed for 10 min to extract nucleic acid. The mixture was centrifuged, and the aqueous phase was collected, and the genomic DNA was precipitated with 500 μL isopropanol. The DNA pellet was washed with 500 μL cold 70% ethanol and air dried, and the DNA pellet was resuspended in 100 μL of DNase-free water. The primers Fungal 18S 5′ UTR fw (GACCTCGGCCCTTAAATAGC) and 3′ UTR rv (CTCGGCCAAGGTGATGTACT) and the mouse glyceraldehyde-3-phosphate dehydrogenase (GAPDH) primers 5′ UTR fw (GAGGGACTTGGAGGACACAG) and 3′ UTR rv (ACATCACCCCCATCACTCAT) were amplified using SYBR green PCR master mix (Thermo Fisher Scientific).

### Docking.

The docking study of SglA was carried out using AutoDock4.2 (41, 42) and followed a five-step protocol of missing-loop construction of SglA: produce the binding site coordinates, create the three-dimensional (3D) molecular formats of hit compounds, parameterize, dock, and evaluate the predicted binding affinities of hit compounds. (i) Since the crystal structure of SglA contains missing loop regions, the Modeller function in UCSF Chimera (43) was used to construct the nonterminal missing loop regions between residues 570 and 627 and residues 669 and 675. The model with the highest discrete optimized protein energy (DOPE) score was chosen for integration with the crystal structure of SglA that was used for docking analysis (44). (ii) As there is no cocrystal structure of SglA with a hit compound, the binding site coordinates of SglA were obtained by overlaying the SglA with the high-resolution cocrystal structure of the Sgl1-Hit 9 complex (PDB code 7LPQ) in UCSF Chimera. These coordinates were then used to create the grid coordinates of hit 9 in SglA in MGLTools (41). (iii) 2D molecular structures of hit compounds were drawn via PerkinElmer’s ChemDraw and saved in MOL format. These coordinates were parsed through the Avogadro molecular editor (45) to generate 3D structures in the biologically relevant protonation state (at pH 7.4) using parameters of the integrated Open Babel tool kit. The internal energy minimization was done with Merck Molecular ForceField (MMFF94) (46–50) until the energy gradient reached approximately 0.0 kJ/mol, and then the coordinates were written in the MOL2 format. (iv) The resulted MOL2 structures were processed through MGLTools to parameterize them for use with AutoDcok4.2 by merging the nonpolar hydrogens, assigning Gasteiger charges, defining rotatable bonds, and writing the output in PDBQT format. (v) Docking calculations were carried out via the Scripps Research Institute AutoDock4.2 program. The default settings of the Lamarckian genetic algorithm (41) were used for the sampling method where 10 solutions were written for evaluation. (vi) Finally, the selection of the representative docking solution was chosen with root mean square deviation (RMSD) less than 2 Å. The visualization and depiction of selected docking results were accomplished through UCSF Chimera/PyMOL molecular visualization programs.

### Statistics.

Statistical analysis was performed using Prism 9 (GraphPad Software) and conducted on data from three or more biologically independent experimental replicates. Data in column graphs are means and standard deviations (SD) from at least three independent experiments, and individual data points are plotted. Statistical significance was analyzed using an unpaired Student’s *t* test for two groups or ordinary one-way analysis of variance (ANOVA) with Dunnett’s multiple-comparison test and one-way ANOVA with Tukey’s multiple-comparison test for multiple groups, with *P* values of <0.05, 0.01, and <0.001 considered significant. Survival curves were analyzed using a log-rank (Mantel-Cox) test.

### Study approval.

Mouse experiments were performed in full compliance with the protocol approved by Stony Brook University (IACUC number 341588) and in compliance with the United States Animal Welfare Act (Public Law 98–198). The experiments were carried out in facilities accredited by the Association for Assessment and Accreditation of Laboratory Animal Care.
