# Supplementary figures and images for: Targeting Sterylglucosidase A to Treat Aspergillus fumigatus Infections
Source: mBio. 2023 Mar 6;14(2):e00339-23. doi: 10.1128/mbio.00339-23 (PMC10128061; doi:10.1128/mbio.00339-23)

## Supplementary Figure 1

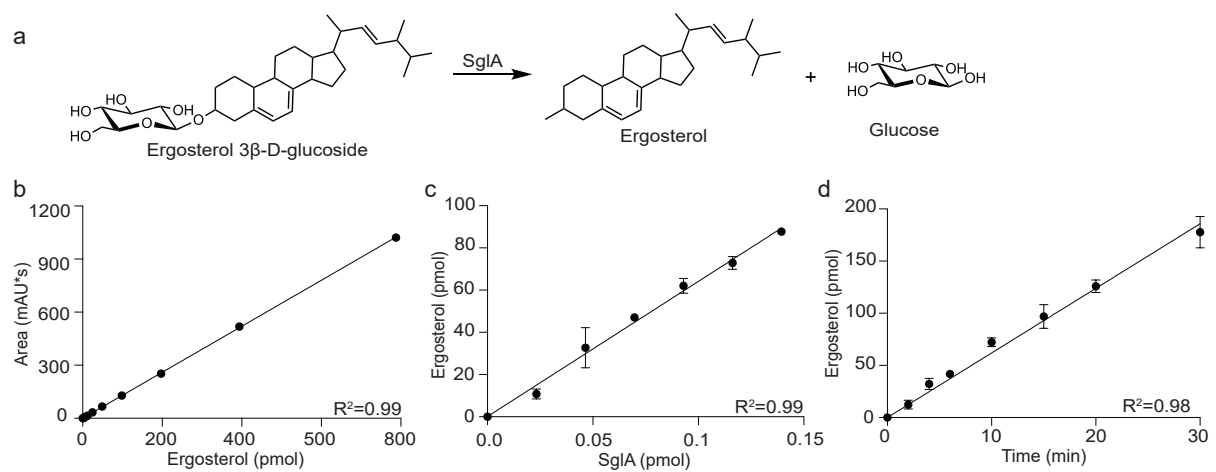

Supplement: FIG S1 [file mbio.00339-23-s0001.pdf]

## Supplementary Figure 2

a

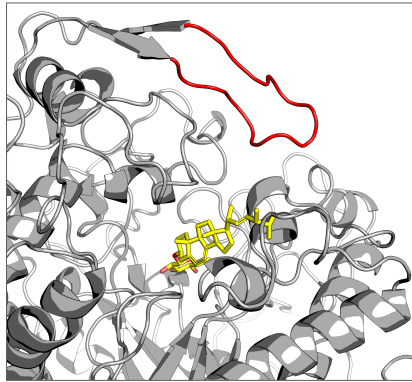

b

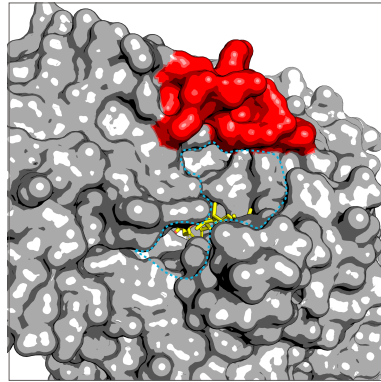

Supplement: FIG S2 [file mbio.00339-23-s0002.pdf]

Supplementary Figure 3

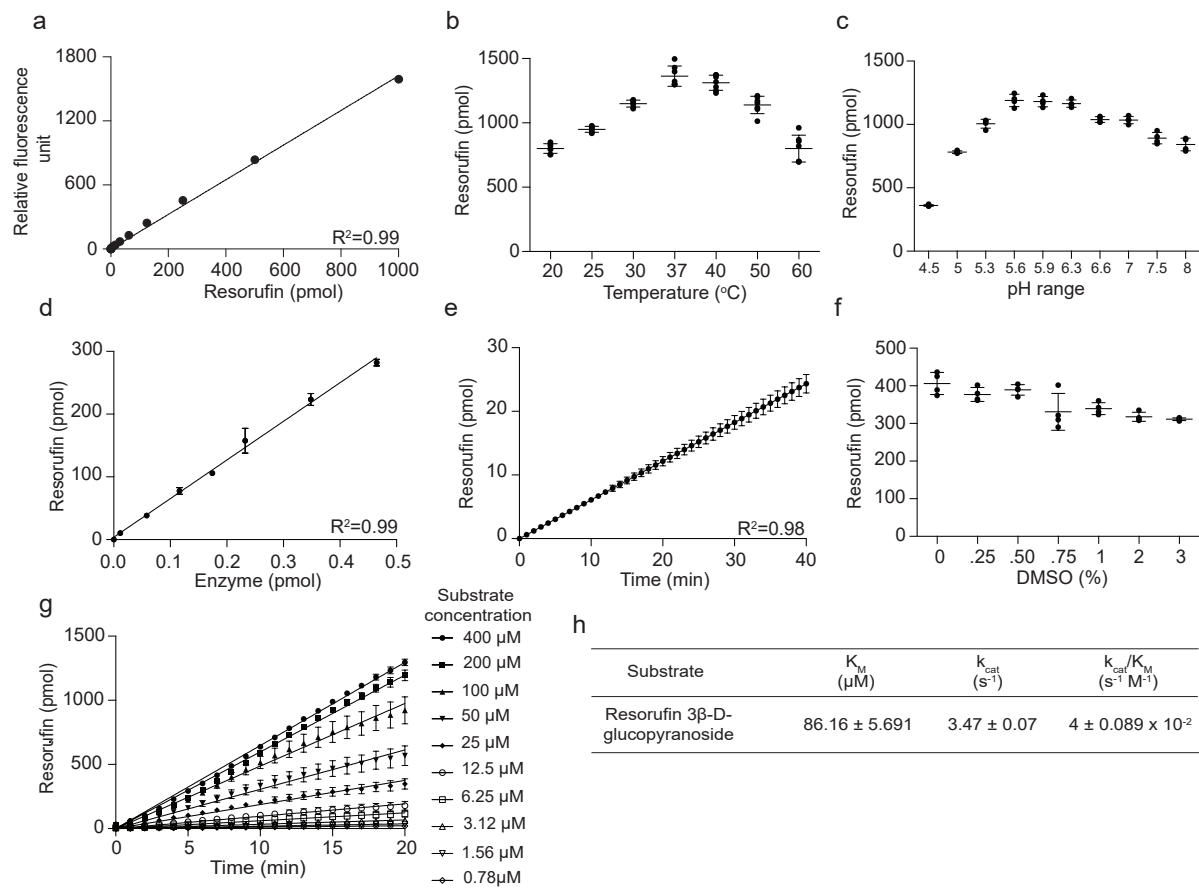

Supplement: FIG S3 [file mbio.00339-23-s0003.pdf]

**Supplementary Figure 4**

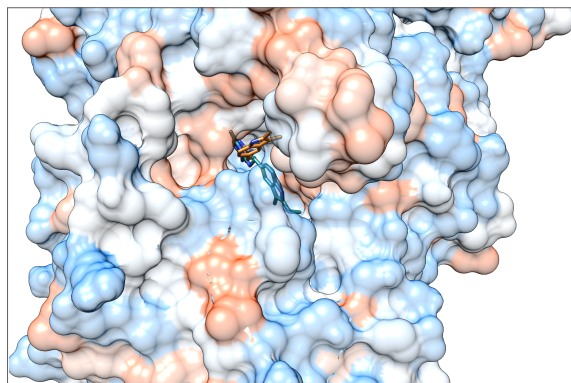

Supplement: FIG S4 [file mbio.00339-23-s0004.pdf]
