# Supplementary material for: Targeting Sterylglucosidase A to Treat Aspergillus fumigatus Infections
Source: mBio. 2023 Mar 6;14(2):e00339-23. doi: 10.1128/mbio.00339-23 (PMC10128061; doi:10.1128/mbio.00339-23)
Supplement: TABLE S1 [file mbio.00339-23-s0005.pdf]

Supplementary Table 1. Small molecule screening data.

Category	Parameter	Value/ Description	Additional information
Assay	Plate format	96-well	Solid black polystyrene microplate, round bottom
	Reaction buffer	pH 6	50 mM citric acid, 176 mM K_2HPO_4, 0.01% Tween-20 and 10 mM sodium taurocholate
	Substrate	10 µL	100 µM stock solution
	Enzyme	17 µL	Containing 20 ng (0.23 pmol) of *Af* SglA
	Incubation time	30 min	37°C
Library	Size	50,000	ChemBridge DIVERSet-CL library
Screen	Cocktail preparation	5000	10 compounds/well at 10 µM each (10% DMSO in DPBS)
	Concentration tested	1 µM	3 µL/well of a 10 µM stock
	Controls	3 µL	Positive control (DPBS) and DMSO control (10% DMSO in DPBS)
	Readout	570 (±10) / 610 (±10) nm	VersaMax™ Microplate Reader
Post-HTS analysis	Cocktails hits criteria	>50% inhibition	Cocktails hits were categorized by percentage of inhibition
	Individuals hits criteria	>50% inhibition	5 µM
	Individual hits confirmation	0.5-4 µM	Dose-response evaluation
	Hit rate	0.04%	20 inhibitors identified from the library
	Additional tests	Toxicity against mammalian cell line A549	Only hits with IC50 ≤2 were evaluated. Hits with selectivity index (SI) higher than 200 were selected
