# Supplementary material for: Targeting Sterylglucosidase A to Treat Aspergillus fumigatus Infections
Source: mBio. 2023 Mar 6;14(2):e00339-23. doi: 10.1128/mbio.00339-23 (PMC10128061; doi:10.1128/mbio.00339-23)
Supplement: TABLE S2 [file mbio.00339-23-s0006.pdf]

Supplementary Table 2. Twenty single compound hits identified in the HTS campaign.

Hits	Chembridge ID	Structure	Mol Formula	Mol Weight	cLogP	IC50 (μM)	LD50 (μM)	SI (LD50/ IC50)
A	13077737	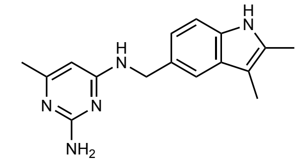	$C_{16}H_{19}N_5$	281.4	3.49	1	40.4	40.4
B	88182154	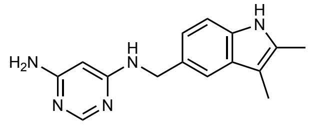	$C_{15}H_{17}N_5$	267.3	1.9	1	350.2	350.2
C	44349962	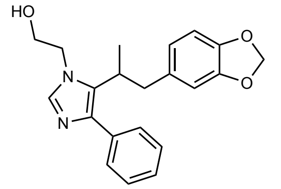	$C_{21}H_{22}N_2O_3$	350.4	3.135	2	1461.2	730
D	38724303	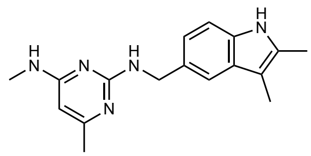	$C_{17}H_{21}N_5$	295.4	2.68	0.5	75.7	151.4
E	70625939	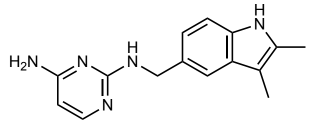	$C_{15}H_{17}N_5$	267.3	1.56	1	181.3	181.3
F	51897881	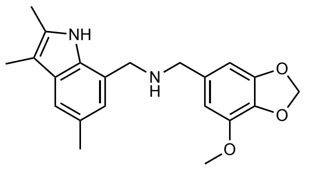	$C_{21}H_{24}N_2O_3$	352.4	3.662	1	NA	-
G	32822315	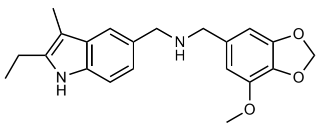	$C_{21}H_{24}N_2O_3$	352.4	3.692	1	31.5	31.5
H	17741735	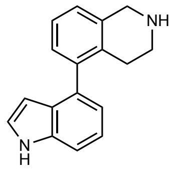	$C_{17}H_{16}N_2$	248.3	3.09	2	NA	-
I	33368230	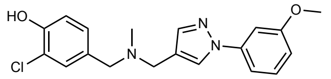	$C_{19}H_{20}ClN_3O_2$	357.8	3.18	1	44.7	44.7

J	20522444	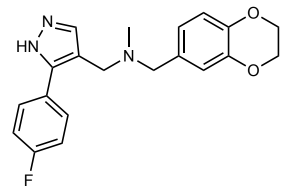	$C_{20}H_{20}FN_3O_2$	353.4	3.753	4	NT	-
K	56254969	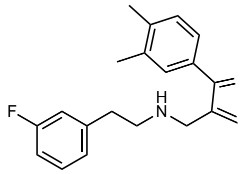	$C_{20}H_{22}FN_3$	323.4	4.595	4	NT	-
L	42450440	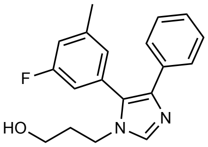	$C_{19}H_{19}FN_2O$	310.4	3.713	4	NT	-
M	34333401	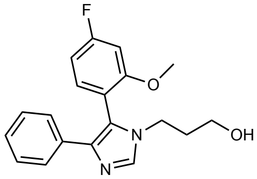	$C_{19}H_{19}FN_2O_2$	326.4	2.742	4	NT	-
N	49876731	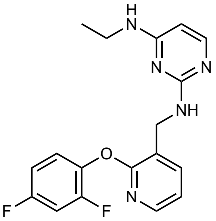	$C_{18}H_{17}F_2N_5O$	357.4	2.41	0.5	22.4	44.8
O	26392226	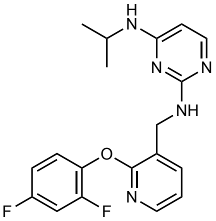	$C_{19}H_{19}F_2N_5O$	371.4	2.76	0.5	19.6	39.2
P	58560626	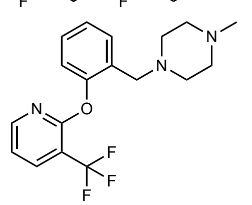	$C_{18}H_{20}F_3N_3O$	351.4	2.53	4	NT	-
Q	34760020	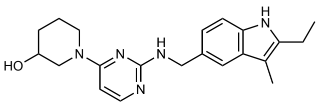	$C_{21}H_{27}N_5O$	365.5	2.39	2	89.7	44.8
R	44071483	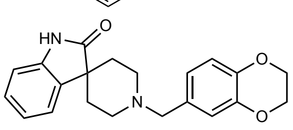	$C_{21}H_{22}N_2O_3$	350.4	2.59	4	NT	-

S	25828831	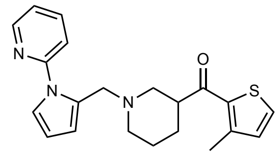	$C_{21}H_{23}N_3OS$	365.5	4.147	4	NT	-
T	98928553	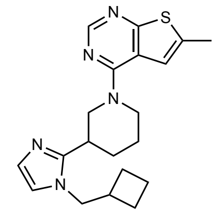	$C_{20}H_{25}N_5S$	367.5	3.35	2	87.1	43.5

NT, toxicity not tested for hits with IC50 > 2μM. NA, compound not available.
