# Supplementary material for: Targeting Sterylglucosidase A to Treat Aspergillus fumigatus Infections
Source: mBio. 2023 Mar 6;14(2):e00339-23. doi: 10.1128/mbio.00339-23 (PMC10128061; doi:10.1128/mbio.00339-23)
Supplement: TABLE S3 [file mbio.00339-23-s0007.pdf]

Supplementary Table 3. Hit B derivatives.

Deriva-tives	Structure	Chembridge ID	Mol Weight	Mol Formula	Mol Name	cLogP	LD50 (μM)
B1	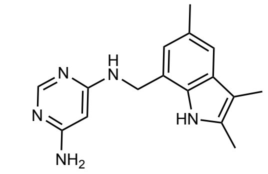	80787085	281.4	$C_{16} H_{19} N_5$	N-[(2,3,5-trimethyl-1H-indol-7-yl)methyl]pyrimidine-4,6-diamine	2.36	ND
B2	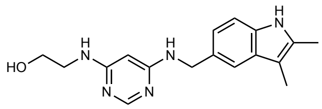	53733165	311.4	$C_{17} H_{21} N_5 O$	2-[(6-{[(2,3-dimethyl-1H-indol-5-yl)methyl]amino}pyrimidin-4-yl)amino]ethanol	3.57	ND
B3	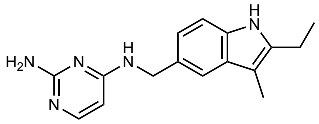	54994060	281.4	$C_{16} H_{19} N_5$	N~4~-[(2-ethyl-3-methyl-1H-indol-5-yl)methyl]pyrimidine-2,4-diamine	3.57	ND
B4	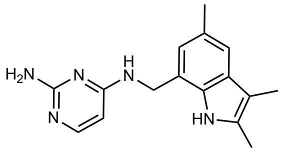	20862167	281.4	$C_{16} H_{19} N_5$	N~4~-[(2,3,5-trimethyl-1H-indol-7-yl)methyl]pyrimidine-2,4-diamine	3.49	ND
B5	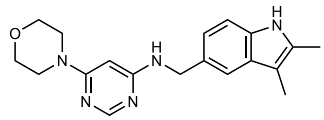	53267402	337.4	$C_{19} H_{23} N_5 O$	N-[(2,3-dimethyl-1H-indol-5-yl)methyl]-6-morpholin-4-ylpyrimidin-4-amine	3.47	ND
B6	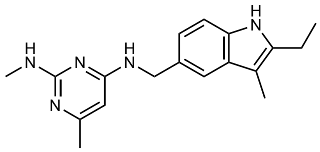	24390922	309.4	$C_{18} H_{23} N_5$	N~4~-[(2-ethyl-3-methyl-1H-indol-5-yl)methyl]-N~2~,6-dimethylpyrimidine-2,4-diamine	2.96	ND
B7	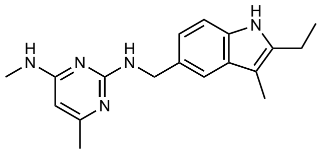	98977858	309.4	$C_{18} H_{23} N_5$	N~2~-[(2-ethyl-3-methyl-1H-indol-5-yl)methyl]-N~4~,6-dimethylpyrimidine-2,4-diamine	3.21	200

ND, not determined.
